# Fine tuning of Rac1 and RhoA alters cuspal shapes by remolding the cellular geometry

**DOI:** 10.1038/srep37828

**Published:** 2016-11-28

**Authors:** Liwen Li, Qinghuang Tang, Takashi Nakamura, Jun-Gyo Suh, Hayato Ohshima, Han-Sung Jung

**Affiliations:** 1 Division in Anatomy and Developmental Biology, Department of Oral Biology, Oral Science Research Center, BK21 PLUS Project, Yonsei University College of Dentistry, Seoul, Korea; 2 Department of Oral Biology, Tohoku University Graduate School of Dentistry, Sendai, Japan; 3 Department of Medical Genetics, Hallym University College of Medicine, Chuncheon, Korea; 4 Division of Anatomy and Cell Biology of the Hard Tissue, Department of Tissue Regeneration and Reconstruction, Niigata University Graduate School of Medical and Dental Sciences, Niigata, Japan; 5 Oral Biosciences, Faculty of Dentistry, The University of Hong Kong, Hong Kong SAR, People’s Republic of China.

## Abstract

The anatomic and functional combinations of cusps and lophs (ridges) define the tooth shape of rodent molars, which distinguishes species. The species-specific cusp patterns result from the spatiotemporal induction of enamel knots (EKs), which require precisely controlled cellular behavior to control the epithelial invagination. Despite the well-defined roles of EK in cusp patterning, the determinants of the ultimate cuspal shapes and involvement of epithelial cellular geometry are unknown. Using two typical tooth patterns, the lophodont in gerbils and the bunodont in mice, we showed that the cuspal shape is determined by the dental epithelium at the cap stage, whereas the cellular geometry in the inner dental epithelium (IDE) is correlated with the cuspal shape. Intriguingly, fine tuning Rac1 and RhoA interconvert cuspal shapes between two species by remolding the cellular geometry. Either inhibition of Rac1 or ectopic expression of RhoA could region-distinctively change the columnar shape of IDE cells in gerbils to drive invagination to produce cusps. Conversely, RhoA reduction in mice inhibited invagination and developed lophs. Furthermore, we found that Rac1 and RhoA modulate the choices of cuspal shape by coordinating adhesion junctions, actin distribution, and fibronectin localization to drive IDE invagination.

Cusps and ridges (also known as crests and lophs), two basic components that are located on the occlusal surface of molars in mammals, generate cuspal diversity by varying their number, size and orientation on the molar crown^1^,^2^.

In general, tooth morphogenesis is a process that is regulated by epithelial-mesenchymal interactions and during which the oral ectoderm thickens, buds, and invaginates to form a cap-like structure, and then a species-specific cusp^3^,^4^. Based on a common morphogenetic concept that a specific shape arises from local differences in cellular behavior regulated by signaling molecules, the cusp formation process involves spatiotemporal changes in cell number, size, shape, and position^5^,^6^. However, many previous studies on cusp formation using mutant mice, including single gene mutants of WNT, FGF, BMP, Notch, and Eda signaling, have focused on the signaling networks that are responsible for the misfolding of the inner dental epithelium (IDE) as well as alterations of cusp patterns^7^,^8^,^9^,^10^,^11^,^12^. Those studies generally clarify the tooth shape based on two principles: the primary enamel knot (PEK) at the cap stage induces secondary enamel knots (SEKs) at the bell stage by a reaction-diffusion model^13^ and, subsequently, SEKs precede future cusps via regionally differential cell proliferation and death in the EK and IDE^14^,^15^. The importance of cellular geometry changes, such as the cell shape, size and growth orientation on the formation of a specific cuspal shape, thereupon the cuspal diversity, remains largely unexplored.

The cell maintains or changes its shape, size and position through the cytoskeleton, cell-cell adhesion, and cell-to-extracellular matrix (ECM) adhesion^16^,^17^,^18^,^19^. Critical regulators of these processes include the Rho family of small GTPases. Among which, Rac1 and RhoA regulate actin filaments (F-actin) into branched networks and cable-like structures, respectively^20^. Much of what we currently know about the roles of GTPases in epithelial morphogenesis has come from studies of invertebrate embryos^21^,^22^, and less information has come from studying models of vertebrate morphogenesis^23^,^24^,^25^. A comprehensive understanding of the cellular geometry that sculpts organ shapes in mammals remains elusive.

Taking advantage of lophodont and bunodont teeth, we revealed that the dental epithelium at the cap stage determines the cuspal shape. In addition to differential cell proliferation, the regionally differential cellular geometry also plays a significant role in the cuspal shaping. We showed that fine tuning of Rac1 and RhoA activities could mediate alternative changes in epithelial invagination by remolding the cellular geometry through the coordination of adherens junctions (AJs), F-actin, and the assembly of the glycoprotein fibronectin (FN) in ECM. Our data provide insight into how the cellular geometry is involved in governing epithelial morphogenesis in tooth development.

## Results

### Cuspal shapes were determined by the dental epithelium at the cap stage

**Figure 1:**
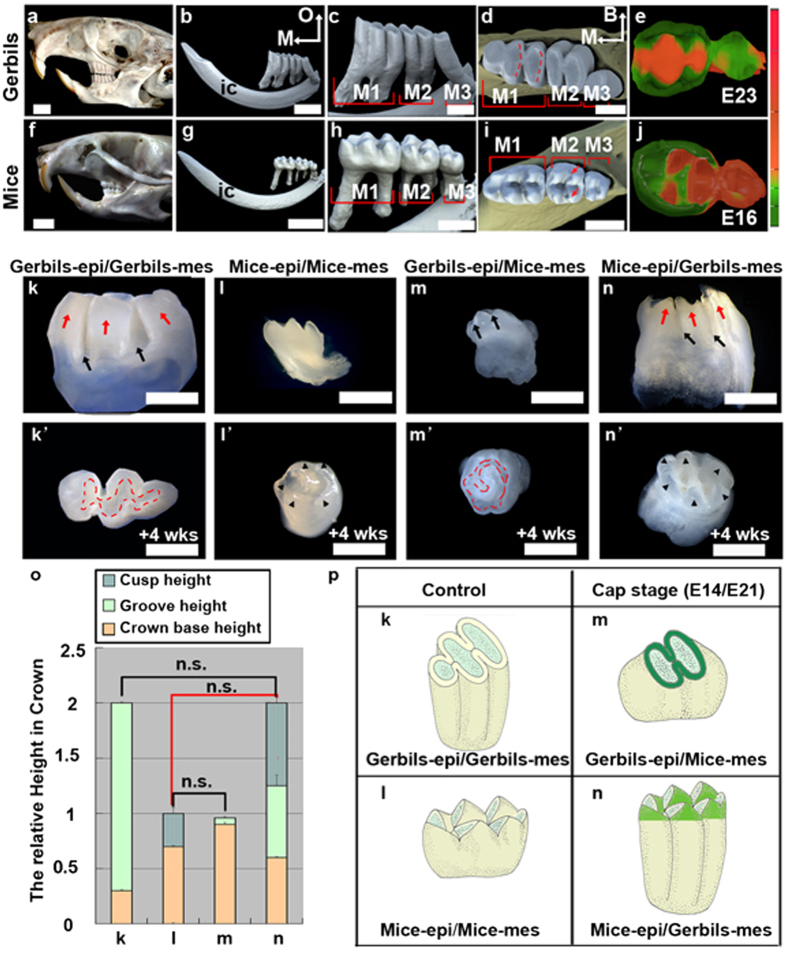
Dental epithelium mediates the cuspal shape at the cap stage. (**a**,**f**) Lateral view (left side) of the skull. (**b**,**c**,**g**,**h**) Lateral view (lingual side), and (**d**,**i**) occlusal view of the right mandible and molars in three-dimensional (3D) images from micro-CT scanning. The red dashed lines indicate the lophs and the arrows indicate the cusp pair. (**e**,**j**) 3D reconstructed images of the dental epithelium at the bell stage viewed from the dental papilla side. The calibration bar is the relative depth of the epithelium. (**k–n**) Lateral view and (**k’–n’**) occlusal view of molar recombinants formed from the indicated components. Red dashed lines indicate lophs, arrowheads indicate cusps, red arrows indicate cylindrical prisms, and black arrows indicate grooves. (**o**) The mean relative heights of cusp (cyan color with waves in the histograms of **l**,**n** in panel o) or grooves (green color in the histograms of **k**,**m**,**n** in panel o) on the crown base (yellow color) for the indicated recombinants in **k**–**n** calculated from 10 teeth per recombinants. The error bars indicate SM. No statistical significance (n.s.) of selected pairwise combinations was indicated by colored lines, black lines represent crown heights (**k**,**n** in panel o) and relative groove heights (**l**,**m** in panel o), and red lines represent relative cuspal heights (**l**,**n** in panel o). (**p**) The schemas show the shape of the cusp and crown for recombinants in (**k**–**n**). M1, first molar; M2, second molar; M3, third molar; M, mesial; O, occlusal; B, buccal; ic, incisor; epi, epithelium; mes, mesenchyme. Scale bar, 4 mm (**a**,**b**,**f**,**g**), 1 mm (**c**,**d**,**h**,**i**), 0.5 mm (**k**–**n**,**k**’–**n**’).

Molars in gerbils (subfamily Gerbillinae, genus *Meriones*) and mice (subfamily Murinae, genus *Mus*) have distinctly different shapes, although they evolved from a common ancestor with Cricetinae dentition^26^,^27^. Gerbil molars have a lophodont pattern (Fig. 1a–d), in which elongated ridges called lophs run between the buccal-lingual cusps, forming approximately flat occlusal surfaces. In comparison, mouse molars are bunodont teeth, with separate cusps (Fig. 1f–i). Both species have similar stages of morphogenesis, despite the different gestation times and molar sizes (Supplementary Fig. S1a–d). Subtle morphological differences were evident at the cap stage. A swollen PEK was morphologically recognized only in mice (Supplementary Fig. S1e,h). At the bell stage, the three-dimensional (3D) shapes of the epithelium reflected species-specific crown shapes (Fig. 1e,j).

Our previous tissue recombination studies revealed that tooth size and cusp size are determined by the mesenchyme and epithelium, respectively^28^. However, it remains unclear whether this principle applies to tooth shape and cuspal shape. We examined the cuspal shapes in hetero-specific tissue recombinants at the cap stage. The crown shape of the teeth in the homo-specific recombinational first molar (M1) of both gerbils (Fig. 1k,k’; N = 9/10) and mice (Fig. 1l,l’; N = 10/10) was the same as that in during development *in vivo*. Lophodont teeth with circle lophs (Fig. 1m’; red dashed lines) and slight grooves on the lateral surface (Fig. 1m; arrows) were generated by recombinants of the gerbil epithelium and mouse mesenchyme (N = 8/10). Reciprocally, bunodont teeth with prominent cusps formed in recombinants of the mouse epithelium and gerbil mesenchyme (Fig. 1n,n’; N = 9/10). Herein, we also observed that six cusps were localized on the periphery of the occlusal surface (Fig. 1n’; arrows) rather than occupying the occlusal surface, as in mouse teeth (Fig. 1l’; arrows), corresponding to the position of three cylindrical prisms (Fig.1n; red arrows) on each lateral surface of gerbil teeth. In addition, the statistical data of the recombinants indicate that the relative cusp height was similar to that of the species that provided the dental epithelium (Fig. 1o; pairs with red lines), and the crown height and groove height were similar to those of the species that provided the dental mesenchyme (Fig. 1o; pairs with black lines). These data demonstrated that the cuspal shape was determined by the dental epithelium at the cap stage (Fig. 1p). Careful examination of apoptosis in the dental epithelium showed that apoptosis was absent in IDE cells in gerbils and was present in those cells in mice (Supplementary Fig. S1f,i). Non-proliferating cells were scattered among IDE cells in gerbil tissue and were restricted in the SEK in mice (Supplementary Fig. S1g,j). Proliferation and apoptosis reflect the local growth rates rather than growth orientations, suggesting that additional cellular events contribute to the invagination of the IDE and cusp formation in mice.

### The cuspal shapes of the molar crown correlated with the cellular geometry of the IDE cells

**Figure 2:**
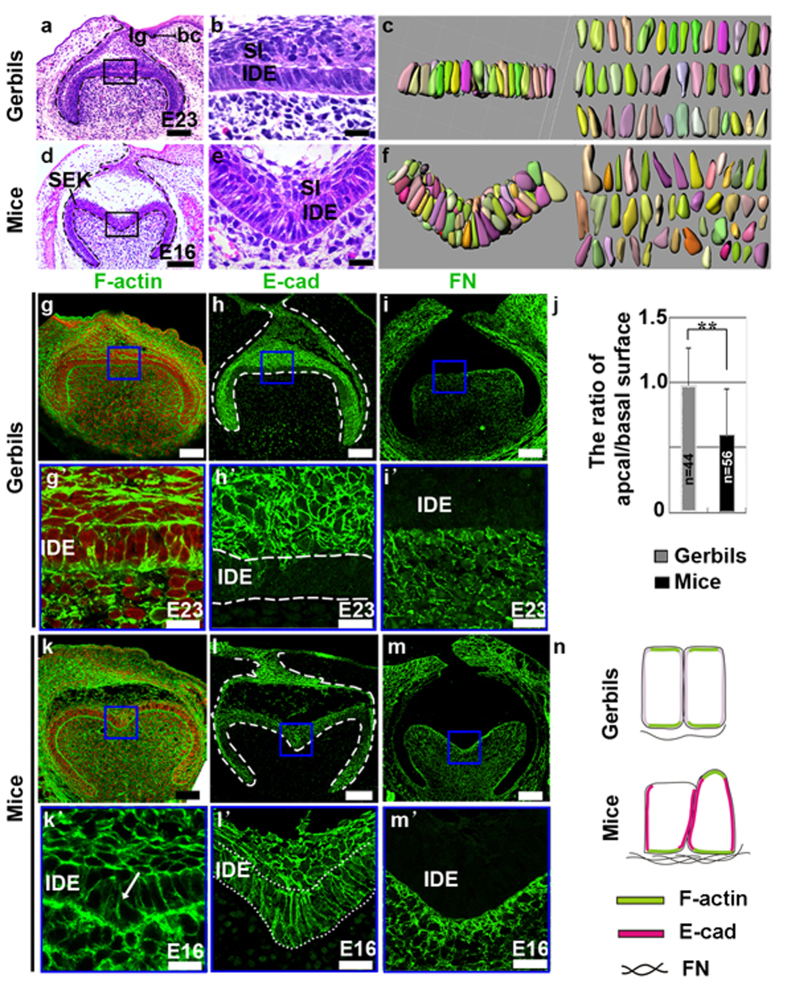
Comparative analysis of cellular geometry in the IDE of gerbils and mice. (**a**,**d**) Frontal sections of the first molar at the bell stage. (**b**,**e**) High magnification images of the black boxes in (**a** and **d**) respectively. (**c**,**f**) 3D reconstructions of individual IDE cells in regions of the panels in (**b** and **e**) after staining with β-catenin to trace the cellular membranes. (**j**) Quantitative measurement of the apical/basal surface ratio of individual cells (**P ≤ 0.01). The error bars indicate SM. (**g**–**i**,**k**–**m**) Staining of F-actin, E-cad, FN in the tooth germ of gerbils (**g**–**i**) and mice (**k**–**m**) at the bell stage. (**g’**–**i’**,**k’**–**m’**) Higher magnification view of the IDE in the intercuspal region in mice and the equivalent region in gerbils, as indicated by the blue boxes in panels g–i,k–m. Comparative analysis of F-actin labeling in the IDE showed that F-actin was not present in the apex of IDE cells that have a wedge shape (**k**’; arrow). Red in (**g**,**k** and **k**’) indicates nuclei. (**n**) Schema comparing the cell shapes and localization of indicated proteins. lg, lingual; bc, buccal; IDE, inner dental epithelium; SI, stratum intermedium; SEK, secondary enamel knots. Scale bar, 100 μm (**a**,**d**,**g**–**i**,**k**–**m**), 20 μm (**b**,**e**,**g**’–**i**’,**k**’–**m**’).

Histological analysis revealed a straight sheet of IDE cells with apically localized nuclei in gerbils (Fig. 2a,b) and folded IDE cells with positioned randomly nuclei in mice (Fig. 2d,e). Because tissue morphogenesis is largely driven by changes in the shapes of individual cells^29^, to obtain 3D cell shapes during cusp formation, we reconstructed the surface rendering for individual IDE cells at the bell stage in gerbils and mice. Nine consecutive frontal sections, each 5 μm thick, were traced after the cell membranes were marked by the cell membrane marker β–catenin. Gerbil IDE cells were columnar in shape (Fig. 2c), whereas mouse IDE cells in the intercuspal region formed wedge-like shapes by minimizing their basal or apical surface (Fig. 2f). Quantitative analysis of the apical and basal surfaces demonstrated that the ratio of the apex to the base in individual IDE cells was 0.98 in gerbils and 0.60 in mice (Fig. 2j). In addition, IDE cells were columnar at the cap stage in gerbils, but PEK and SEK cells in mice were cuboidal. These observations demonstrated that the cuspal shape correlates with the cellular geometry. Given that the changes in the shape of individual cells could drive tissue morphogenesis^29^, we characterized IDE cells in detail. Intriguingly, cytoskeletal F-actin strongly localized at both the apex and base in gerbils (Fig. 2g,g’). Additionally, F-actin was present at the base of the IDE cells in mice. However, the distribution of F-actin was inconsistent at the apex of different IDE cells (Fig. 2k,k’). E-cadherin (E-cad), a marker of AJs, was weak in IDE cells of the occlusal region in gerbils (Fig. 2h,h’). In mice, E-cad was present in IDE cells of the intercuspal regions (Fig. 2l,l’). FN was evenly distributed in mesenchyme in gerbils (Fig. 2i,i’) and was particularly enriched in the mesenchyme surrounding the invaginated epithelium (Fig. 2m,m’). These observations suggest that the local levels of F-actin and E-cad and the spatial assembly of FN correlate with the apical or basal width of IDE cells (Fig. 2n), which caused the cuspal shapes to correlate with the cellular geometry.

### Fine regulation of Rac1 and RhoA alters cuspal shapes

**Figure 3:**
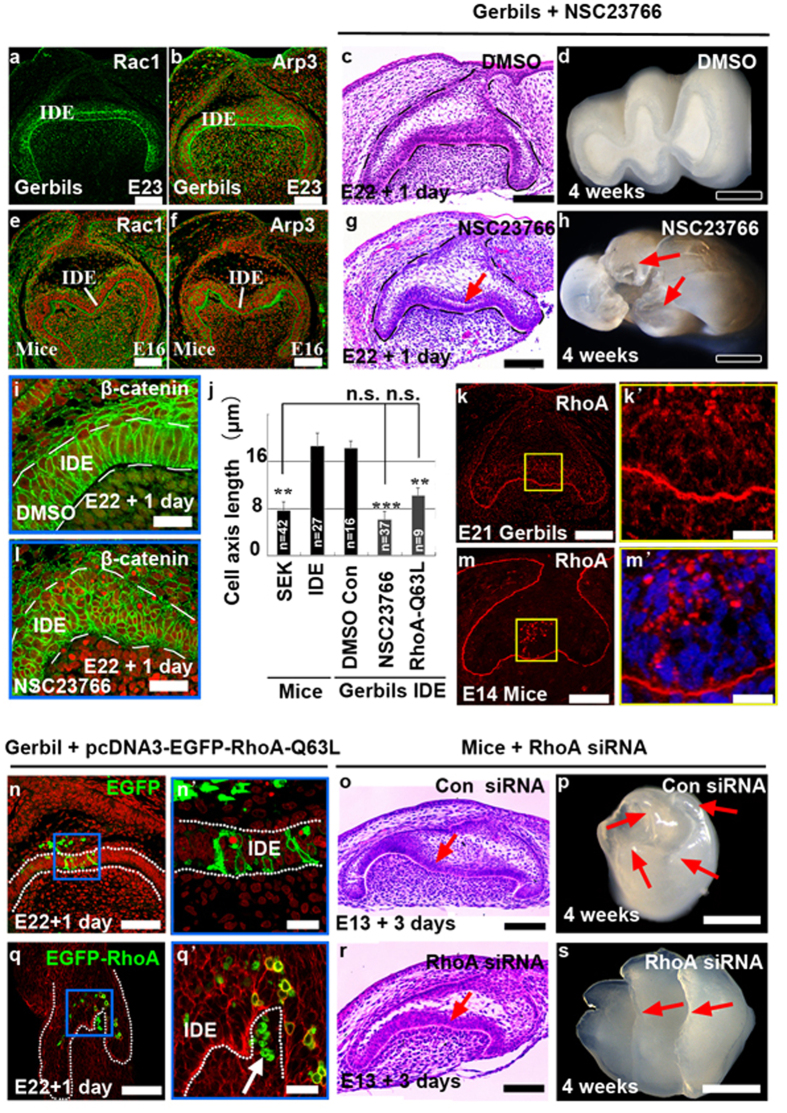
Rac1 and RhoA mediate epithelial invaginations and cuspal shapes. (**a**,**b**,**e**,**f**) Localization of Rac1 and Arp3 in the first molar in gerbils and mice at the bell stage. (**c**,**d**,**g**,**h**) Phenotypes of gerbil tooth germs and teeth after treatment with the DMSO and NSC23766 (90 μM in DMSO). The arrow in panel g indicates ectopic invagination of IDE, and the arrows in panel h indicate cusps. (**i**,**l**) Localization of β-catenin in the cuspal regions in the tooth germ with or without NSC23766 shows cell shapes and sizes. TO-PRO-3 was used as counter staining. (**j**) The cell length of the SEK in mice and IDE cells in gerbils with NSC23766 or pcDNA-EGFP-RhoA-Q63L were dramatically shortened when compared with the length of the IDE in mice and in gerbil controls (**P ≤ 0.01; ***P ≤ 0.001). n.s., not significant. The error bars indicate SM. (**k**,**m**) Localization of RhoA in the first molar in gerbils and mice at the early cap stage. (**n**,**n’**,**q**,**q’**) The formation of a pit (**q’**; arrow) and changes of cell shapes after electroporation of pcDNA-EGFP-RhoA-Q63L (**q**,**q’**) compared to electroporation with pcDNA-EGFP vehicles (N, N’) in IDE cells. (**n’**,**q’**) Higher magnification images of the blue boxes in panel n and q. (**o**,**p**,**r**,**s**) Phenotypes of tooth germs (**o**,**r**) and teeth (**p**,**s**) after treatment with control siRNA and RhoA siRNA (300 nM). Arrows in panel o and r show the loss of invagination of IDE by RhoA siRNA. Arrows in panel p and s indicate the loss of cusps and the formation of ridges by RhoA siRNA. Stages are indicated in each panel. IDE, inner dental epithelium. Scale bar, 500 μm (**d**,**h**,**p**,**s**), 200 μm (**n**,**q**), 100 μm (**a**–**c**,**e**–**g**,**k**,**m**,**o**,**r**), 20 μm (**i**,**l**,**k’**,**m’**,**n’**,**q**).

The ultimate cuspal shape is produced by epithelial invagination, a process requires the changes of cell motility, cell adhesion, and cellular shape. Because all of these cellular behaviors can be regulated by Rac1 and RhoA^30^, it is not difficult to reason that Rac1 and RhoA are involved in establishing cuspal shape-correlated IDE cellular geometry during cusp formation. To verify this, we first examined the localization of cytoskeleton-related Rac1 and actin-related protein 3 (Arp3) in molars at the bell stage. Interestingly, Rac1 and Arp3 were highly localized at the apex and base of IDE cells in gerbils (Fig. 3a,b), whereas their distribution was high at the base but weak at the apex in mice (Fig. 3e,f). Together with the localization of F-actin, E-cad, and FN in IDE cells of both species (Fig. 2g–m,g’–m’), our results implied that Rac1 is involved in establishing the specific cell shape of IDE by regulating the distributions of F-actin, E-cad, and FN.

Intriguingly, when a widely used specific Rac1 inhibitor, NSC23766m, was applied to E22 gerbil tooth germs, inhibition of Rac1 activity induced ectopic invaginations, reminiscent of intercuspal regions in the mice (Fig. 3g), while treatment with DMSO had no effect on tooth morphology (Fig. 3c). One pair of buccal-lingual cusps was evident in calcified teeth after NSC23766 treatment (Fig. 3h; red arrows, *n* = 5/10), in contrast to lophodont teeth in the control (Fig. 3d; *n* = 10/10). Correspondingly, NSC23766 treatment markedly changed the cell shape (Fig. 3i,l). Quantitatively characterizing the cell shape by measuring the cell length showed that NSC23766-induced compact-cuboidal IDE cells in the cuspal region (Fig. 3l) had an average length of 6 μm (Fig. 3j), resembling SEK cells, which have an average length of 7 μm (Fig. 3j). Conversely, control IDE cells were columnar (Fig. 3i), with an average length of 18 μm (Fig. 3j).

Interestingly, RhoA, an antagonist of Rac1 regarding the determination of cell shape, was remarkable in PEK cells in mouse molars at the early cap stage (Fig. 3m,m’), but minimally enriched in gerbil molars (Fig. 3k,k’). Therefore, we manipulated RhoA activity by electroporation of constitutively active pcDNA-EGFP-RhoA-Q63L into IDE cells in E22 gerbil tooth germs. Increased RhoA activity induced a pit in IDE cells, leading them to become compact-cuboidal in shape (Fig. 3q,q’), whereas ectopic expression of the EGFP construct had no effect on cell or tissue morphogenesis (Fig. 3n,n’). RhoA-Q63L-expressing cells were 50% shorter than average compared with the control (Fig. 3j). Conversely, we reduced the RhoA level in the E13 mouse tooth germ by treatment with 300 nM siRNA. These tooth germs showed impaired IDE invagination (Fig. 3r,s). Furthermore, calcified teeth after transplantation showed that some buccal-lingual cusps were fused and formed ridges after RhoA siRNA treatment (Fig. 3s; red arrows, *n* = 4/10). In the control, E13 mouse tooth germs treated with control siRNA initiated the development of cusps after 3 days culture (Fig. 3o) and produced cuspidated teeth (Fig. 3p; *n* = 10/10). Manipulation of Rac1 and RhoA could govern the invagination of the IDE, thus generating a variation of cuspal shape in teeth. These results reveal the critical roles of cellular geometry in tooth morphogenesis.

### The remolding of the cellular geometry during the alteration of cuspal shapes

**Figure 4:**
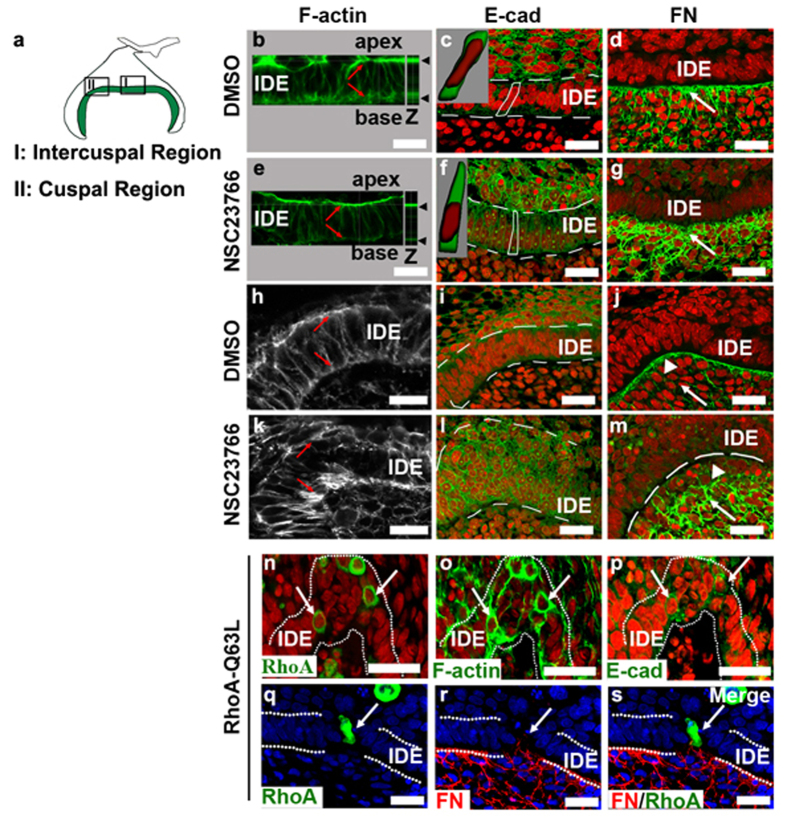
Remolding of the cellular geometry in the IDE cells during GTPase-mediated epithelial invagination. (**a**) Schema indicates the regions of intercusps (**I**) and cusps (**II**), from which the images were taken for (**b**–**g** and **h**–**m**) respectively. (**b–m**) Effects of NSC23766 on F-actin, E-cad and FN in the IDE of gerbils in intercuspal regions (**b**–**g**) and in cuspal regions (**h**–**m**). Red indicates the nuclei. (**b**,**e**,**h**,**k**) Equal levels of F-actin assembled in the apex and base of IDE cells (**b**,**h**; arrows) were disrupted (**e**,**k**; arrows) after NSC23766 treatment. **Z** in panel b and e indicates the vertical view. (**c**,**f**,**i**,**l**) E-cad was slightly increased both in the cuspal and intercuspal regions after NSC23766 treatment. The graphic images of cytoplasm (*green*) and nucleus (*red*) inserted in panel c and f are 3D reconstructions of the outlined cells, showing the cell shapes and nuclear localization. The cells had a triangular shape in the intercuspal region and a round-compact shape in cuspal regions. (**d**,**g**,**j**,**m**) The unique assembly of FN in the basement membrane along the IDE sheet was disrupted by NSC23766. The localization of FN increased both in the intercuspal (**g**; arrow) and cuspal regions (**m**; arrow) compared with the control (**d**,**j**; arrows), but lost its assembly in the basement membrane in the cuspal regions (**m**; arrowhead). The formation of a pit in the IDE after several IDE cells overexpressed EGFP-RhoA-Q63L by electroporation. Green fluorescence indicates individual cells expressing RhoA-Q63L (**n**). RhoA-Q63L-expressing cells (**n**) had enhanced expression of F-actin (**o**) and E-cad (**p**) compared with neighboring cells. (**q–s**) FN (red) deposited around the side of RhoA-Q63L-expressing IDE cells (arrows). Blue indicates nuclei. White dashed lines outline the IDE. IDE, inner dental epithelium. Scale bar, 20 μm.

To understand how cellular geometry alterations drove epithelial invaginations, we examined the effects of NSC23766 and pcDNA-EGFP-RhoA-Q63L on the localization of F-actin, E-cad and FN in the gerbil, three important components of cellular geometry. The alterations of cellular geometry by NSC23766 were spatially dependent. In IDE cells in intercuspal regions, F-actin was slightly reduced in the apex and was dramatically disrupted in the base by NSC23766 (Fig. 4e) compared with F-actin in the DMSO-treated tooth germ (Fig. 4b). The expression levels of E-cad increased, making IDE cells wedge-like in shape with a basal localization of the nuclei after NSC23766 treatment (Fig. 4c,f). FN deposition also increased, compared to the control (Fig. 4d,g). In the cuspal regions, NSC23766 treatment disrupted the symmetrical assembly of F-actin in the apex and base of IDE cells (Fig. 4h,k). IDE cells were depolarized and became compact-cuboidal in shape after NSC23766 treatment, as showed by the increased expressions of E-cad (Fig. 4i,l). While the assembly of FN was observed in the basement membrane in the DMSO-treated tooth germ (Fig. 4j; arrowhead), NSC23766 treatment caused a loss of FN in the basement membrane (Fig. 4m; arrowhead) and its increase in the adjacent mesenchymal cells (Fig. 4m; arrow). These results demonstrated that NSC23766 functions in a region-specific manner to drive epithelial invagination, by which it remodels cell shape in the intercuspal regions and simultaneously induces cell compaction in the cuspal regions. On the other hand, the ectopic activity of RhoA (Fig. 4n) also caused compaction of IDE cells with increased expression of F-actin (Fig. 4o) and E-cad (Fig. 4p). Impressively, FN was re-deposited around RhoA-Q63L-expressing IDE cells (Fig. 4q–s), switching the epithelial cell adhesion from cell-cell contacts to cell-ECM interactions. It reveals that FN plays a critical role in the orientation of epithelial growth.

**Figure 5:**
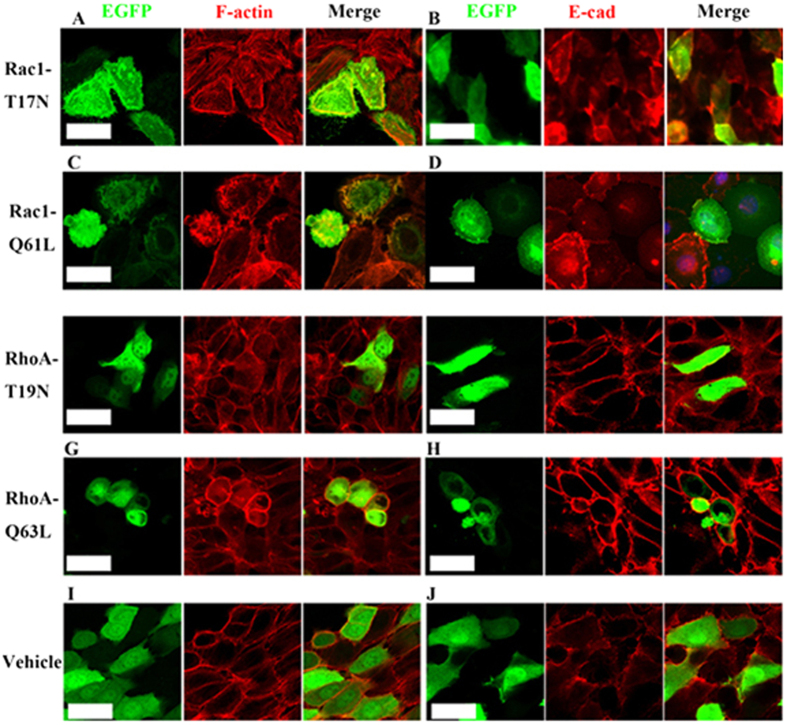
Rac1 and RhoA regulate the cell geometry in the IDE cells *in vitro*. (**a–j**) Visualization of dental epithelial cells transfected with dominant negative Rac1-T17N (**a**,**b**), constitutively active Rac1-Q61L (**c**,**d**), dominant negative RhoA-T19N (**e**,**f**), constitutively active RhoA-Q63L (**g**,**h**), and vehicle control constructs (**i**,**j**), followed by staining with F-actin (red) and E-cad (red). Scale bar, 20 μm.

To further clarify how the Rac1 and RhoA activities regulate cell shape and size of individual cells, dental epithelial cells were transfected with Rac1-T17N (dominant-negative Rac1), Rac1-Q61L (constitutively active Rac1), RhoA-T19N (dominant-negative RhoA), or RhoA-Q63L (constitutively active RhoA) that were inserted into pcDNA3-EGFP backbone vector. Dominant negative Rac1-T17N reduced branched F-actin (Fig. 5a), but upregulated E-cad (Fig. 5b). Consistently, constitutively active Rac1-Q61L increased branched F-actin (Fig. 5c), but reduced E-cad (Fig. 5d). However, the effects of Rac1 on the cell shape and cell size of individual cells were variable, reminding us of the observations from the Trowell cultures that Rac1 had region-specific effects on IDE cells. Dominant negative RhoA-T19N slightly reduced cable-like F-actin (Fig. 5e) and had few effects on either the expression level of E-cad (Fig. 5f), or cell shape and cell size (Fig. 5e,f). Conversely, constitutively active Rac1-Q61L remarkably increased cable-like F-actin (Fig. 5g) and E-cad (Fig. 5h) expression, as well as the compaction of cells (Fig. 5g,h). Transfection of empty vectors had no any effects (Fig. 5i,j). The consistency of the effects of Rac1 and RhoA on the assembly of F-actin, expression level of E-cad, and cell shape and cell size either in Trowell culture or in cultured individual cells suggested that the cell shape and cell size were direct outcomes of Rac1 and RhoA rather than the consequence of IDE invagination.

**Figure 6:**
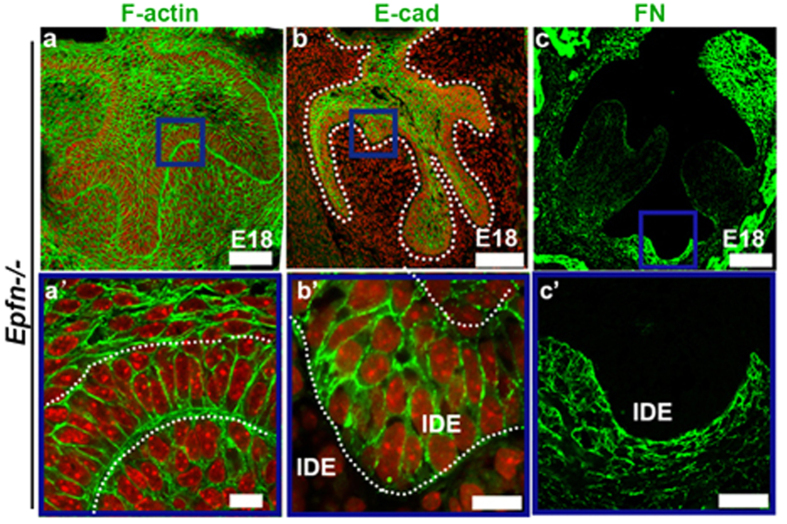
Localizations of F-actin, E-cad, and FN in the multiple epithelial invaginations in the IDE of *Epfn−/−* mice. (**a–c**,**a’–c’**) The frontal sections of *Epfn−/−* mice at the bell stage stained with E-cad, F-actin and FN. (**a’–c’**) High-magnification images of the blue boxes in (**a–c**). F-actin (*green*) was intensely distributed in the base of IDE cells, whereas accumulation of F-actin in the apical margin of the IDE cells was absent (**a**,**a’**). E-cad was faint in IDE cells of the cuspal regions in *Epfn−/−* mice (**b**), but was intense in IDE cells in the intercuspal regions (**b’**). Less deposition of FN was observed in the basement membrane in the cuspal regions (**c**), whereas intensive deposition of FN was observed in the basement membrane surrounding the invaginated IDE (**c’**). IDE, inner dental epithelium. Scale bar, 100 μm (**a**–**c**); 20 μm (**a’**–**c’**).

To determine whether the participation of the cellular geometry in tooth morphogenesis in gerbils and mice is a common feature of epithelial invagination, we examined the localization of E-cad, F-actin and FN in multiple branching dental epithelia, a phenotype found in the *Epiprofin−/− (Epfn−/−*) mouse^31^,^32^. We detected a biased distribution of F-actin at the basal margin of IDE when the epithelium is folded (Fig. 6a,a’) and found that E-cad was enriched in IDE cells of the intercuspal regions and cervical loop, but was faint in the cuspal region (Fig. 6b,b’). In addition, FN was deposited in the basement membrane around invaginated epithelium (Fig. 6c,c’). Collectively, our results demonstrated that alterations of the orientation of epithelial growth require proper Rac1 and RhoA activities, which remold cellular geometry through coordination of the polarization of F-actin in the apexes and bases of IDE cells, the spatial distribution of E-cad and local deposition of FN in invaginated regions.

## Discussion

**Figure 7:**
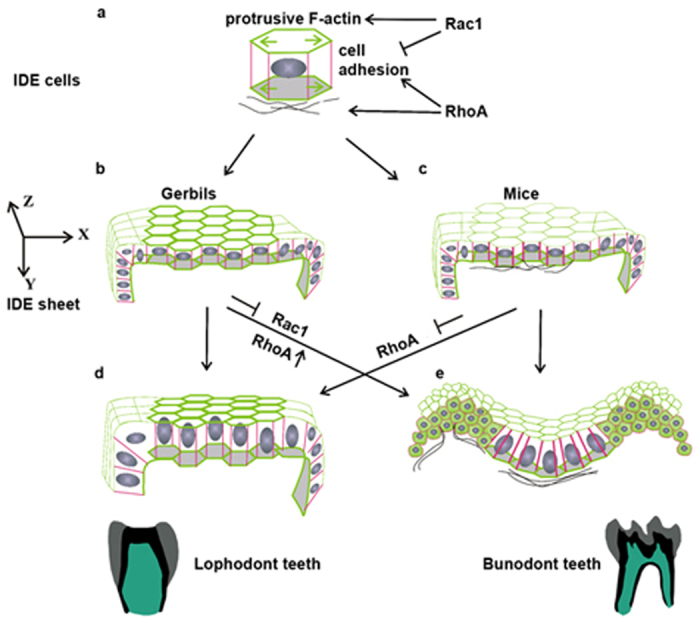
Fine tuning of Rac1 and RhoA altered cuspal shape through the remolding of cellular geometry in the IDE. (**a**) A proposed model showing Rac1 and RhoA control of the cytoskeleton (F-actin), AJs (E-cad), and FN deposition in IDE cells. (**b**,**c**) Different distributions of F-actin, E-cad, and FN are present in IDE cells of gerbils and mice. **X**, **Y**, **Z** indicate the axes in three-dimensional space. (**d**,**e**) Schematic IDE sheets show that Rac1 and RhoA participate in tissue morphogenesis and cuspal shapes by changing the cell shapes, sizes, and FN assembly. IDE, inner dental epithelium.

Omnivorous animals have bunodont teeth, and species with increasing specialization for grazing have lophodont teeth; however, other species have intermediate teeth shapes between these two types^2^. The mechanisms underlying the evolutionary changes in tooth shape could be determined by comparing the developmental processes between different species. By comparing two typical tooth shapes, bunodont teeth of mice and lophodont teeth of gerbils, we report that the cuspal shape is determined by the dental epithelium at the cap stage. This observation could partially explain why the disruption of FGF-mediated crosstalk between the epithelium and mesenchyme by *Sprouty* deficiency, which is mainly expressed in the dental mesenchyme, had no effect on the cuspal shape^33^. The interpretations for the origin of multicusped teeth in mammals are based on two main and rather contradictory theories. The first one is the differentiation theory, which states that multicusped teeth developed from one simply shaped tooth of mammals, whereas the second one is the concrescence theory, which indicates the integration of several primordia of simple teeth that were inherited from mammalian ancestors^34^. Recent studies on molars and incisors provide evidence of a bias for the concrescence theory as the explanation for the origin of multicusped teeth^35^,^36^. Based on the concrescence theory, the lophs in gerbils are equivalent to the fused crests from a pair of buccal-lingual cusps in mice (Fig. 7d,e). The transformation between cusps and lophs presented in our study suggests an ancestral homology of lophs in gerbils and cusp-fused crests in mice, indirectly consolidating the causal relationship between the integration extent and morphological diversity of rodent teeth during evolution.

Researchers who study tooth development generally pay attention to the functions of signaling molecules, such as Wnts, FGFs and TGFβs/BMPs, in regulating cell growth and apoptosis. However, our findings suggest that cell shape, cell size, and growth orientation should also be taken into consideration to understand how signaling molecules act on cellular behaviors to drive tissue morphogenesis. Our results showed a causal link between the cellular geometry and cuspal shape, in which uniformity reflect lophs and un-uniformity reflect cusps. This is perfectly illustrated by our ability to produce cusps from lophs by interrupting the uniformity of the cellular geometry of an IDE sheet. By modulating the activity of RhoA or Rac1, we forced columnar IDE cells in gerbils to adopt a wedge shape in the intercuspal region and a compact-cuboidal shape in cuspal regions. Together with the spatial assembly of FN, IDE sheets in gerbils consequently became equivalent to mouse IDE sheets (Fig. 7b,e). Here, we demonstrated that the ectopic invagination of IDE cells in gerbils is the consequence of cell shape changes. This is consistent with a mathematic model, which describes that various epithelial cell shapes are generated by the coordination of F-actin, AJs and ECM^37^. The compact-cuboidal cell shape present in the cuspal region may allow the epithelium to bend, which is analogous to the acceleration of epithelial invagination in the *Drosophila melanogaster* tracheal placode by the rounding of mitotic cell^18^. Moreover, local mutations that affect the actin belt cause a transition from columnar to the cuboidal cell, inducing additional folds^29^. We speculate that the equal distribution of Rac1-dependent F-actin in the apex and base of IDE cells appears to serve as a protrusive force (Fig. 7a; green arrows) and maintains the columnar shape, resisting invagination of IDE in gerbils (Fig. 7b). The existence of such resistance is supported by the results of perturbation experiments using NSC23766, a widely used inhibitor of Rac1 activation, in which columnar IDE cells in gerbils change into a wedge shape and lose F-actin, either apically or basally, in the intercuspal region (Fig. 7b,e). Previous studies have suggested the possibility that the Rac1-dependent F-actin might exhibit stress stiffening *in vitro*^38^ and can promote cell elongation *in vivo*^24^, and that Rhou, a Cdc43-related atypical Rho GTPase, can affect the distribution of F-actin for mechanical resistance^25^. By contrast, the reduction of F-actin induced by NSC23766 in our study did not reduce the basal width possibly because of counteractions in the movement of the nuclei towards the base (Fig. 4c,f; inserted boxes).

The crucial roles played by Rac1, RhoA, and actin cytoskeleton dynamics in the regulation of E-cad have been broadly studied^39^,^40^. E-Cad-based adhesions can alter the physical aspects of cells such as the stiffness^41^,^42^, spreading^43^ and migration^44^ of single cells, and facilitate the propagation of force in the tissue^45^,^46^. The NSC23766-induced or RhoA-Q63L-induced appearance of E-cad in IDE cells in gerbils likely propagates the intracellular force across intercellular contacts in tissues. Although the patterns of F-actin and E-cad in IDE cells in gerbils after NSC23766 treatment are not exactly the same with mice, which implicates the existence of some other regulatory factors within individual IDE cells to determine the position of F-actin accumulation and to regulate the E-cad dynamics. Nevertheless, the treatment of NSC23766 is sufficient for the invagination of IDE sheet in gerbil, suggesting that Rac1 activity is required for F-actin accumulation and E-cad based AJs to affect the plasticity of the IDE.

FN assembly plays a critical role in IDE invagination. FN assembles in all of the invaginated sites of IDE in wild type and *Epfn−/−* mice. Disruption of basement membrane adhesion either by the mutation of *laminin α5* or reduction of integrin α6 in the tooth germ induces defects in cusp formation in mice^47^. Inhibition of Rac1 and activation of RhoA induced either outward or inward movement of the IDE sheet associated with the spatial assembly of FN (Fig. 7e), especially the presence of FN between two epithelial cells (Fig. 7e). We propose that at the invaginated site, cytoskeleton-associated proteins activate assembly of FN in the basement membrane. The fibrillar FN forms bundles that serve as tracks for the growth of epithelial cells. This mechanism is analogous to a report that FN assembly induces a pit for epithelial branches in the submandibular gland or lung^48^. It has been shown that overexpression of RhoA in the thyroid epithelial cells can promote FN assembly^49^, which might through the interaction with transmembrane integrin receptors^50^. These results suggest that the cuspal shape is controlled by the epithelium in concert with the basement membrane or underlying mesenchyme. In addition, RhoA is critical for the condensation of mesenchymal cells in early tooth development^51^. The enrichment of RhoA on the tip of the enamel organ in the mouse at the cap stage might also contribute to the condensation of PEK.

There were multiple prerequisites to teeth evolution in mammals. Current study shows that, after primary enamel formation, the dental epithelium patterns the cusp shape. Cell shape, size and orientation of growth, which are regulated by cytoskeleton-associated proteins Rac1 and RhoA, are involved in IDE invagination and morphological diversity in molars. We report that Rac1 and RhoA regulate cellular geometries by affecting the localization of F-actin, E-cad, and FN (Fig. 7a). Cell and tissue shapes have plasticity and are able to transform between different shapes. We speculate that the evolution of cusp patterns arose from extrinsic environmental forces rather than an intrinsic property of cells. The activities of GTPases may be defined by spatial distributions of growth factors signaling from EK. Comparative studies on inter-specific nucleotide variation suggest that the evolution of cusp patterning might be attributed to the evolutionary changes in enhancers, such as Fgf9ECR1 and Fgf10ECR3, which affect the expression of major signaling molecules involved in tooth development^52^. The evolved enhancers are associated with positively selected genes, which occur under environmental changes^53^.

While these studies have increased our understanding of cusp patterning during evolution, they may also provide important models to address critical questions towards the integration of gene expression, cellular geometry and tissue morphogenesis in the future.

## Materials and Methods

### Animals

Institute of Cancer Research (ICR) strain mice were purchased from Koatech Technology Corporation (Koatech Co, Korea). Mongolian gerbils were maintained at the Department of Oral Biology, Yonsei University College of Dentistry. Embryonic samples of *Epfn−/−* knock-out mice were prepared in the Department of Oral Health and Development Sciences, Tohoku University Graduate School of Dentistry. All experiments were performed according to the guidelines of the Yonsei Animal Use Committee (#LML08-272), and all of the experimental protocols for animal research were approved by the Intramural Animal Use and Care Committee of Yonsei University College of Dentistry (Permit Number: 2193).

### Immunofluorescence (IF) and 3D reconstruction of cells

Antibodies against the following antigens were used: Ki-67 (Spring Bioscience), E-cadherin (R&D Systems), β-catenin (NeoMarkers Biotechnology), fibronectin (BD Biosciences), RhoA and Rac1 (Cell Signaling Technology), and Arp3 (Sigma). Phalloidin-FITC (Invitrogen) and TUNEL assays (Trevigen, MD) were performed following the manufacturer’s instructions. Images were acquired on a Zeiss LSM700 meta-confocal laser-scanning microscope. To show the 3D reconstruction of cells, surface rendering was performed semi-automatically using the contour drawing model in Imaris 7.1. To quantitatively analyze the surfaces of the apex and base as well as the length of IDE cells, β-catenin outlines were manually traced using the measurement model in Imaris 7.1.

### Heterospecific recombination

Cap stage tooth germs were used for heterospecific recombination. We separated the dental epithelium and mesenchyme with Dispase (Gibco) and recombined them as heterospecific-interacting components. The recombinants were transplanted under kidney capsule for 4weeks after Trowell culture for 2 days.

### Plasmids construction and transfection

EGFP-containing plasmids expressing RhoA-Q63L, RhoA-T19N, Rac1-Q61L, and Rac1-T17N were obtained from Addgene. Molar primary epithelial cells were obtained from the molars of E14.5 mice. Lipofectamine^®^ 2000 (Invitrogen) was used for gene delivery. After transfection of plasmid DNA for 2 days, cells were harvested for IF.

### Application of inhibitors, electroporation and Trowell culture

NSC23766 (Millipore) was dissolved in H_2_O and diluted in DMEM with Glutamax (Gibco) and 10% FBS to obtain a final concentration of 90 μM. A total of 300 nM RhoA-siRNA (Santa Cruz Biotechnology) was delivered by Lopofectamine^®^ 2000 (Life Technologies) reagent according to the manufacturer’s instructions.

For electroporation, Fast Green (Sigma, 1:1000) was added to both of the plasmid solutions of pcDNA-EGFP-RhoA-Q63L and pcDNA-EGFP for visualization within the tissue. A microcapillary needle was used to inject 1 μg/μL DNA into the dental epithelium of gerbil molars at E22.0, followed by four pulses at 40 V applied using an electroporator for 50 milliseconds with 1 second intervals.

Mandibular molar regions were obtained from E22.0 gerbil and E13.0 mouse embryos. Molar explants were cultured *in vitro* using the Trowell technique. Ten specimens were examined via the morphological and IF analyses.

## Additional Information

**How to cite this article**: Li, L. *et al*. Fine tuning of Rac1 and RhoA alters cuspal shapes by remolding the cellular geometry. *Sci. Rep.* **6**, 37828; doi: 10.1038/srep37828 (2016).

**Publisher's note:** Springer Nature remains neutral with regard to jurisdictional claims in published maps and institutional affiliations.

## Supplementary Material

Supplementary Information
