# Supplementary material for: Fine tuning of Rac1 and RhoA alters cuspal shapes by remolding the cellular geometry
Source: Sci Rep. 2016 Nov 28;6:37828. doi: 10.1038/srep37828 (PMC5124948; doi:10.1038/srep37828)

Title: Fine tuning of Rac1 and RhoA alters cuspal shapes by remolding the cellular geometry

Authors:

Liwen Li[1, a],

Qinghuang Tang[1, a],

Takashi Nakamura[2],

Jun-Gyo Suh[3],

Hayato Ohshima[4],

Han-Sung Jung[1, 5] *

[a] ***Both authors contributed equally to this paper.***

Affiliations:

[1] Division in Anatomy and Developmental Biology, Department of Oral Biology, Oral Science Research Center, BK21 PLUS Project, Yonsei University College of Dentistry, Seoul, Korea;

[2] Department of Oral Biology, Tohoku University Graduate School of Dentistry, Sendai, Japan;

[3] Department of Medical Genetics, Hallym University College of Medicine, Chuncheon, Korea;

[4] Division of Anatomy and Cell Biology of the Hard Tissue, Department of Tissue Regeneration and Reconstruction, Niigata University Graduate School of Medical and Dental Sciences, Niigata, Japan;

[5] Oral Biosciences, Faculty of Dentistry, The University of Hong Kong, Hong Kong SAR.

***Corresponding author:**

Han-Sung Jung: hsjung@yuhs.ac

Figure S1: Comparison of the molar development between gerbils and mice.

(**a**) Developmental stages of odontogenesis in the lower first molar. Mouse embryonic day 0 (E0) was designated as the day on which a vaginal plug was detected. Vaginal smears were used to assess the estrus cycle of the female gerbils. The females were mated if the results of the vaginal smear indicated either pre-estrus or estrus between 1700 and 1800 hours. The following day was counted as E0. (**b**) The embryonic day is specified by the wet body weight. (**c**, **d**) Occlusal views of tooth germ in gerbils and mice at the cap and bell stage. (**e**, **h**) Histology of a transversely sectioned M1 at the cap stage. PEK indicates a swollen structure in the dental epithelium of mice. (**f**, **g**, **i**, **j**) TUNEL staining and Ki-67 immunostaining of the transversely sectioned M1. The black boxes in each panel present high magnification images of IDE on the occlusal surface of gerbils and in the intercuspal regions of mice. The arrows show the non-proliferating cells; the red dashed lines indicate the border between IDE and SI cells. The stages are indicated in the left corner of each panel. IDE, inner dental epithelium; SI, stratum intermedium. Scale bar, 300 μm (**c**, **d**), 100 μm (**e**, **h**), 200 μm (**f**, **g**, **i**, **j**).

Figure S1: Comparison of the molar development between gerbils and mice.

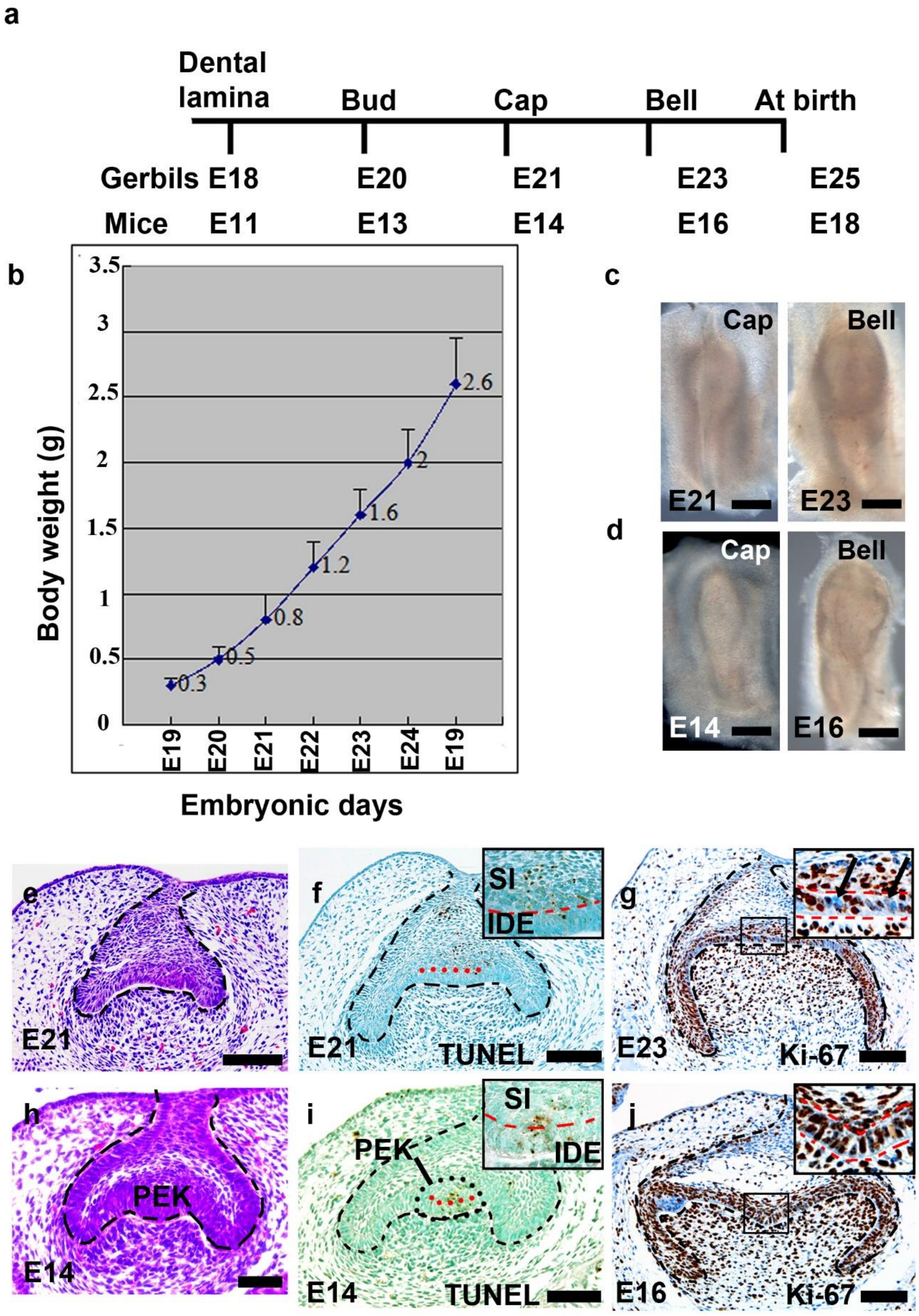

Supplement: Supplementary Information [file srep37828-s1.pdf]
